# Bioorthogonal Click Chemistry Enables Stable Cell Attachment to Silk Fibroin Scaffolds for Tissue Engineering Applications: Proof of Concept in Neural Regeneration

**DOI:** 10.1021/acs.biomac.6c01009

**Published:** 2026-08-26

**Authors:** Ana Quintana-Prego, Atocha Guedan-Duran, Gustavo Victor Guinea, Juan Gomez-Rivas, Fivos Panetsos

**Affiliations:** † Center for Biomedical Technology, Universidad Politécnica de Madrid, Pozuelo de Alarcón, 28223 Madrid, Spain; ‡ Silk Biomed SL, 28260 Madrid, Spain; § Neurocomputing and Neurorobotics Research Group, Faculty of Biology and Faculty of Optics, 16734 Complutense University of Madrid, 28040 Madrid, Spain; ∥ Bioactive Surfaces SL, 28260 Madrid, Spain; ⊥ Biomaterials and Regenerative Medicine Group, Instituto de Investigación Sanitaria del Hospital Clínico San Carlos (IdISSC), 28040 Madrid, Spain; # Department of Material Science, ETSI Caminos, Canales y Puertos, Universidad Politécnica de Madrid, 28040 Madrid, Spain; ∇ Biomedical Research Networking Center in Bioengineering, Biomaterials and Nanomedicine (CIBER-BBN), Instituto de Salud Carlos III, 28029 Madrid, Spain; ○ Urology Service, Instituto de Investigación Sanitaria del Hospital Clínico San Carlos (IdISSC), 28040 Madrid, Spain; ◆ Omnia Mater SL, 28009 Madrid, Spain; ¶ Human Retina SL, 28009 Madrid, Spain

## Abstract

Tissue engineering seeks effective strategies to integrate cells into scaffolds while preserving spatial organization and stability. Conventional approaches based on extracellular matrix molecules or adhesion peptides often provide weak and nonspecific attachments, limiting robust assembly of complex cellular architectures. Here, we investigate biorthogonal click chemistry to address these limitations. By enabling covalent cell-biomaterial bonding through strain-promoted azide–alkyne cycloaddition (SPAAC), we achieved stable cell-scaffold integration. As a proof of concept, we engineered artificial Bands of Büngner-like scaffolds by functionalizing silk microfibers with cyclooctyne groups and metabolically engineering Schwann cells to present azide functionalities. Over 15 days *in vitro*, these biohybrid scaffolds supported directed neurite outgrowth, preserved metabolic activity, and increased nerve growth factor levels at early time points. Our results support click chemistry as a robust strategy for assembling stable cell-laden scaffolds while preserving biological function, potentially enabling the tailored design of complex cellular architectures and microenvironments for tissue engineering applications.

## Graphical abstract

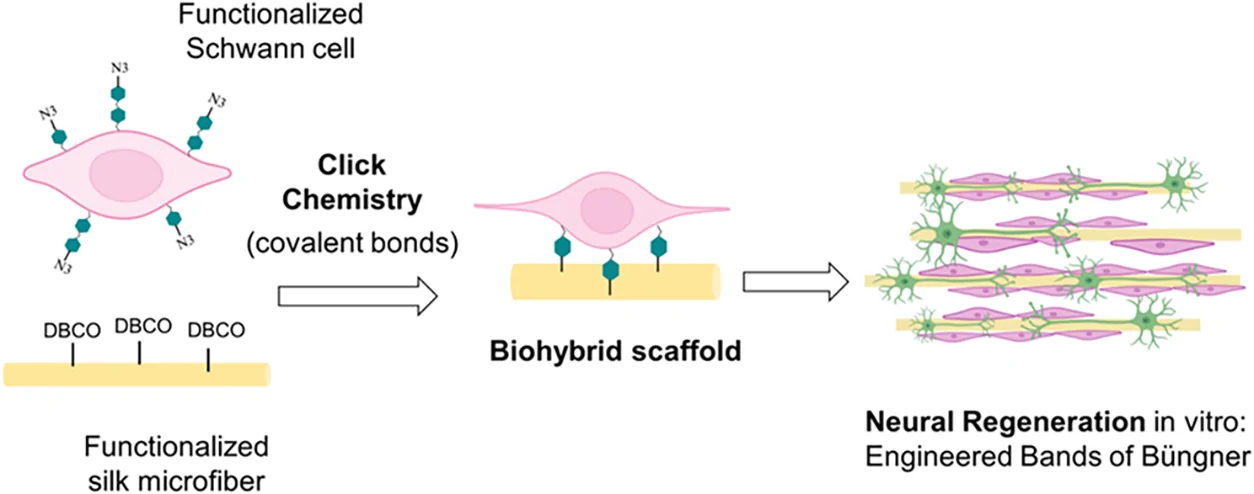

## 1 Introduction

Tissue engineering has as its main goal the development of artificial tissues that not only replace and repair damaged areas but also improve the regeneration of the host’s own tissues, aiming to restore both the structure and functionality of the injured organ.^1^ The most commonly applied strategies, due to their easy translatability to the clinic, are based on the use of decellularized matrices or biocompatible materials in various formats,^2^ such as hydrogels, meshes, or conduits, that serve as physical scaffolds for the infiltration of native cells. However, the low efficiency of these treatments highlights that topographical cues alone are insufficient to achieve tissue regeneration, and chemical signals are also needed to stimulate the process.^3–7^ This has led to the development of more complex strategies that attempt to replicate both the physical and chemical cues involved in the regeneration process. To this end, the incorporation of cells into artificial scaffolds has been extensively investigated,^2^ as they contribute to the establishment of defined molecular microenvironments and enable essential cell–cell interactions.

In many cases, the selection of biomaterials is based on their mechanical properties or adaptability to specific configurations, rather than on their suitability for cell adhesion, and they often require enhancement to promote cellular attachment.^8^ The most common option is using macromolecules derived from the extracellular matrix (ECM), such as collagen, fibronectin, or laminin, as well as adhesion peptides like RGD.^8,9^ However, ECM-derived molecules have several drawbacks, such as the lack of cell selectivity that causes nonspecific interactions, the risk of immunogenicity, or the variability in composition and purity.^9–15^ Moreover, ECM-derived molecules offer noncovalent binding sites that are susceptible to modulation by microenvironmental factors after implantation in the damaged tissue, including fluctuations in pH, ionic strength, and enzymatic activity.^16–18^ These weak interactions enable cell migration within the scaffold, but this feature may become disadvantageous when the aim is to establish stratified cellular layers or complex tissue architectures. Considering all of these factors, noncovalent interactions represent a relatively limited and sometimes insufficient approach to achieving effective cell-scaffold integration.

By contrast, covalent conjugation involves the formation of stable, irreversible bonds and represents a promising strategy to overcome the limitations of noncovalent interactions. Because covalent interactions are relatively uncommon in natural cell–cell adhesion, their use to improve cell attachment to biomaterials has not been extensively explored. In this context, click chemistry reactions offer a family of simple, efficient, and highly selective reactions that yield covalent bonds under physiological conditions.^19,20^ Their high specificity prevents undesired reactions with hydroxyl or amine groups in proteins, thereby minimizing the formation of side products. Click chemistry, first introduced by Sharpless et al. in 2001,^19^ was later adapted by Bertozzi et al.^20^ to develop copper-free click reactions, which are biocompatible and suitable for covalent functionalization of biological systems. Among these, strain-promoted azide–alkyne cycloaddition (SPAAC) involves the reaction between an azide and a strained cyclooctyne to form a stable triazole ring. SPAAC has been widely used for targeted imaging,^21–25^ drug delivery,^26–28^ glycoproteomic analysis,^29–31^ or cell therapy.^32,33^ However, its application in tissue engineering is more limited. Liu et al.^17^ used SPAAC ligation to covalently conjugate human neural progenitor cells (NPCs) to collagen fibers for spinal cord repair, observing an enhancement in NPCs attachment, differentiation, and axonal growth, while Mao et al.^33^ achieved a specific and long-lasting infiltration of DBCO-modified films with N3-macrophages *in vivo*. Motivated by these promising results and by the scarcity of tissue-engineering studies using this approach, we explored the potential of SPAAC to create robust and durable biohybrid scaffolds for nerve regeneration.

As a proof of concept, we engineered artificial Bands of Büngner (BoB) by combining Schwann cells (SCs) with aligned silk microfibers. After nerve injury, SCs have a crucial role in creating longitudinal cell structures called Bands of Büngner that provide the physical substrate to guide growing axons to the distal nerve stump.^34–36^ In addition, SCs secrete neurotrophic factors and present contact-mediated cues that further support neuroregeneration.^34–38^ To enable covalent binding, we functionalized the SC surface with azide groups using metabolic glycoengineering. This technique was previously applied by Au et al.,^39^ who functionalized mouse Schwann cells (SCs) with PD-L1 and CD68 *via* click chemistry to develop a cell therapy for multiple sclerosis. However, the potential of the click reactions to enhance the adhesion strength between SCs and biomaterials has not yet been explored. Here, SCs were incubated with tetraacetylated N-azidoacetyl-D-mannosamine (Ac4ManNAz), which is metabolized by the cells, resulting in azide groups displayed on membrane glycoproteins and glycolipids. In parallel, silk fibroin microfibers were fabricated and functionalized with dibenzocyclooctyne (DBCO), which reacts specifically with azides to form covalent bonds. We then combined azide-modified SCs with DBCO-modified microfibers to generate artificial BoB and assessed both cell integration and scaffold robustness. Finally, we assessed the biological performance of these biohybrid scaffolds *in vitro* to confirm that covalent cell adhesion does not compromise scaffold functionality.

## 2 Materials and Methods

### 2.1 Production and Characterization of Silk Scaffolds

A solution of SF was obtained using the protocol previously described by Madurga et al.^40^ The first step was the degumming of the cocoons to remove the silk sericin coating of the SF microfibers. For this, the cocoons were cut into small fragments of around 2 or 3 cm^2^ up to a total of 5 g. One liter of distilled water was boiled with Na_2_CO_3_ (Sigma-Aldrich, 222321) (0.2% by weight), and cocoon fragments were added. After 1 min, it was stirred, allowed to settle for 5 min, and thereafter it was stirred every 5 min for a total of 20 min. The liquid was removed, and the SF was washed with distilled water. It was left to dry at 60 °C for 4 h. Then, SF was dissolved in a solution of 9.2 M LiBr (Thermo Scientific Chemicals, 013408.36) (10% by weight) in distilled water. Dialysis was performed using a membrane with a 3.5 kDa cut-off to remove excess salt. The water was alternately changed every 8 and 16 h for 3 days. The final mixture was centrifuged at 5000 rpm for 30 min to remove any debris. Finally, reverse dialysis consisted of a PEG 8000 Da (MP Biomedicals, 194839) solution with 1 M CaCl_2_ (Sigma-Aldrich, 21102), which was performed for 24 h to obtain a solution of SF. It was stored at 4 °C for later use.

High-performance regenerated silk fibroin (RSF) microfibers were produced using Dynamic Dope Destabilization Spinning (D3S). The flow rate of the SF dope was 5 μL/min, and the flow rate of the focusing fluid was 50 mL/h. Microfibers were collected directly on glass coverslips (Thermo Scientific, Waltham, MA, USA) of 15 mm diameter fixed on a take-up mandrel that rotated at a speed of 15 cm/s. Once the microfibers were spun, they were glued by their ends to the coverslip using DOWSIL TM 738 Electrical Sealant (Dow). Two scaffold formats were generated: one with aligned microfibers and another with disorganized microfibers in random orientations. Microfiber density was controlled during fabrication by adjusting the spinning time, while fiber orientation was defined by the collection setup. Both formats were produced with comparable microfiber densities. For its use in cell cultures, microfibers were sterilized in the oven at 130 °C for 45 min and stored at room temperature.

### 2.2 Topography and Cytotoxicity Studies of RSF Microfibers

Scanning Electron Microscopy (SEM) images of the RSF microfibers were obtained to study their surface topography. First, individual microfibers were attached to a specific SEM support and then metalized using a Cathodic Sputtering Metallizer (Q150R Plus, Quorum Technologies). Subsequently, SEM images of the microfibers were acquired with a Hitachi S-3000N scanning electron microscope at a magnification of 1000×. The diameter of 21 microfibers was measured using ImageJ software, and an average value was calculated.

To evaluate the cytotoxicity of the biomaterial, a CyQUANT XTT Cell Viability Assay (X12223, Invitrogen) was performed. RSF meshes were first sterilized by autoclaving at 121 °C for 20 min and subsequently incubated in primary culture medium at a concentration of 2.5 mg SF/mL for 24, 48, or 72 h at 37 °C. After each incubation period, the biomaterial was removed, and the conditioned media were collected and stored at −20 °C until further use. SCs were seeded into 96-well plates at a density of 1 × 10^4^ cells per well. After 24 h, the culture medium was replaced with 200 μL of conditioned medium previously incubated with the RSF meshes, and cells were cultured for an additional 24 h. As a positive control, cells were maintained in fresh culture medium that had not been exposed to the biomaterial. Following the exposure period, the medium was removed and replaced with a reaction mixture consisting of 24.9 μL of XTT reagent, 4.2 μL of electron-coupling reagent, and 70.8 μL of culture medium per well. Cells were then incubated for 4 h at 37 °C in the dark. Finally, absorbance was measured at 450 nm using a microplate reader.

### 2.3 Mechanical and Physicochemical Characterization of RSF Microfibers

The mechanical properties of the RSF microfibers were evaluated by tensile testing. Individual microfibers were mounted on paper foil frames with a 10 mm gap length. The microfibers were photographed in three different regions under an optical microscope, and the average diameter was measured using ImageJ 1.53t software for subsequent mechanical analysis. The paper foil frames containing the individual microfibers were then mounted on a pair of mechanical testing clamps. The entire assembly was submerged in distilled water and placed on a Precisa XT 2020A balance (Precisa Gravimetrics AG). Tensile tests were carried out at a crosshead speed of 1 mm/min using an Instron 4411 mechanical tester. During the tests, weight and time data were collected and processed to obtain true stress–true strain curves, assuming volume conservation. From these curves, the maximum stress, elastic modulus, and work of fracture were determined. The elastic modulus was calculated as the slope of the curve in the initial elastic region, and the work of fracture was obtained as the area under the true stress–true strain curve using the trapezoidal rule.

For physicochemical characterization, crystallinity changes were evaluated by analyzing the secondary structure of RSF microfibers before and after 1, 2, and 3 months of immersion in distilled water at RT. Attenuated Total Reflectance Fourier Transform Infrared Spectroscopy (ATR-FTIR) was performed on wet samples. Spectral data were processed using OMNIC software to quantify the contribution of β-sheet, α-helix, random coil, and turn structures. Peak deconvolution was performed using the following parameters: Gaussian peak fitting, medium sensitivity, full width at half height (FWHH) of 0.964, noise target of 0.1, and linear baseline correction. Peak assignments of the amide I region were performed according to the vibrational band assignments for *Bombyx mori* SF reported by Hu et al.^41^ (Table 1).

**Table 1:** Assignment of Infrared Absorption Bands within the Amide I Region According to Wavenumber (cm^–1^) and Their Corresponding Secondary Structural Conformations in *B. mori* SF

wavenumber (cm^–1^)	secondary structure
1610–1620	Aggregated β-strand/Intermolecular β-sheet
1621–1627	Intermolecular β-sheet
1628–1637	Intermolecular β-sheet
1638–1655	Random coil
1656–1662	Helical structures
1663–1670	β-turn
1671–1694	β-turn
1696–1703	Intermolecular β-sheet

### 2.4 Scaffold Conditioning for Click Chemistry

RSF microfibers were functionalized for copper-free click chemistry reaction using dibenzocyclooctyne-*N*-hydroxysuccinimidyl ester (DBCO-NHS) (Sigma, 761524). Microfibers collected into coverslips were incubated with DBCO-NHS (0.016 mg DBCO/mg SF) and *N*,*N*-diisopropylethylamine (DIPEA) (0,3 μL DIPEA/mg FS) in 1× PBS. Microfibers were incubated at room temperature overnight. Then, they were washed 3 times in 1× PBS with 0.01% Tween 20 (Sigma, P1379) and stored at 4 °C. DBCO-functionalized microfibers were labeled with FAM (fluorescein) azide (Lumiprobe, 35130). Nonfunctionalized microfibers were used as negative controls. Microfibers were incubated with FAM azide in DMSO in a proportion of 2 mol FAM azide: 1 mol DBCO, for 2 h at room temperature. Then, microfibers were washed 3 times in 1× PBS with 0.5% Triton X-100 (Sigma, T8787).

Images of the microfibers were taken at 20× magnification using a Leica DMI3000 B inverted microscope equipped with A4 BP360/40, L5 BP480/40, and N2.1 BP 515–560 filters and a high-resolution digital camera attached to the microscope. Fluorescence intensity of nonfunctionalized (*n* = 4 individual microfibers) and DBCO-functionalized microfibers (*n* = 11 individual microfibers) was quantified using ImageJ software. Fluorescence intensity was measured in Regions of Interest (ROIs) of constant area placed on the microfiber surface, and ROI values from the same microfiber were averaged to obtain one value per microfiber for statistical analysis.

The correct functionalization of microfibers with DBCO was verified by spectroscopy. First, 4 mg of RSF microfibers were incubated with 0.32 mg/mL DBCO-NHS and DIPEA in 1× PBS overnight with gentle shaking. The next day, the DBCO-microfibers were washed twice in 1× PBS with Tritón 2% and twice in 1× PBS and dissolved in 200 μL of 9.2 M LiBr to obtain a homogeneous solution. As controls, nonfunctionalized microfibers were dissolved. The UV–vis spectra of DBCO-microfibers and nonfunctionalized ones were obtained between 250 and 400 nm using a UV–vis Biodrop Duo+. The standard curve of DBCO-NHS was used to calculate the concentration of DBCO groups in the DBCO-microfibers. The amount of DBCO per mg of SF was calculated with eq 1

1 $$\mathrm{DBCO}\;\mathrm{density}\;\left(\mathrm{nmol}\;\mathrm{DBCO}/\mathrm{mg}\;\mathrm{SF}\right)=\frac{C_{\mathrm{DBCO}}\cdot V\cdot 10^{3}}{{\mathrm{MW}}_{\mathrm{DBCO}}\times m_{\mathrm{FS}}}$$

where *C*_DBCO_ is the concentration of bound DBCO (μg/mL), *V* is the solution volume (mL), MW_DBCO_ is the molecular weight of DBCO (202.25 g/mol), and *m*_SF_ is the mass of silk fibroin (mg).

### 2.5 Cell Cultures

Two cell lines were used for this research. Primary rat Schwann cells derived from the spinal nerves of postnatal day 8 rats were purchased from Innoprot (P10301) and cultured at 37 °C and 5% CO_2_ in a commercial Schwann Cell Medium Kit (Innoprot, P60123) composed of 500 mL of Schwann Cell basal medium, 25 mL of Fetal Bovine Serum (FBS), 5 mL of Schwann Cell Growth supplement and 5 mL of penicillin/streptomycin (P/S) solution. Cells were grown in P100 plates until 80% confluent. For coculture studies, a Mouse Motor Neuron-like Hybrid (NSC-34) cell line (Tebubio, CLU140) was used. NSC-34 cells were cultured in Dulbecco’s Modified Eagle Medium (DMEM) with 4.5 g/L d-glucose supplemented with 10% FBS (10100, Gibco), 1× l-glutamine (Gibco, 25030–024), and 1× P/S (Gibco, 15140122) at 37 °C and 5% CO2. Cells were grown in P100 plates until 80% confluent.

### 2.6 SCs Conditioning for Click Chemistry

SCs were cultured at a density of 6 × 10^4^ cells per well in a P48 multiwell plate and incubated at 37 °C and 5% CO_2_ for 24 h. After this time, the medium was removed and N-azidoacetylmannosamine-tetraacylated (Ac4ManNAz) (Sigma, 900917) was added to fresh media. Concentrations of 20 μM and 40 μM of Ac4ManNAz were used, and cells were incubated for 24, 48, and 72 h.

To check the correct functionalization of the cell surface with azide, azide-SCs were labeled by SPAAC reaction using a Cyanine3 DBCO dye (Lumiprobe, B10F0). Medium was removed, and cells were fixed with 4% paraformaldehyde (PFA) and washed 3 times with 1× PBS. Then, a solution of 10 μM Cyanine3 (Cy3) DBCO in DMSO was added for 30 min with gentle shaking. Finally, cells were washed 3 times with DMSO and 2 times with 1× PBS.

Fluorescence microscopy images were acquired with a Leica DMI3000 B fluorescence microscope. Fluorescence intensity was measured with ImageJ, and statistical comparison was performed between different azide-sugar concentrations and controls for each incubation time to establish the best functionalization conditions.

We evaluated the cytotoxicity of Ac4ManNAz in SCs by indirect study of the cell proliferation capacity with a Cell Proliferation Kit XTT (PanReac, A8088). As previously said, SCs were incubated with 20 and 40 μM Ac4ManNAz for 24, 48, and 72 h. A solution of 2.5 mL of XTT reagent and 0.05 mL of electron-coupling reagent was prepared. 100 μL of specific medium for each cell type and 50 μL of the previously prepared XTT mixture were added to the cells. Cells were incubated for 4 h at 37 °C and 5% CO2. After this time, the plate was gently shaken, and the spectrophotometric absorbance was measured using a microplate reader (ELx800, BioTek). The wavelength to measure the absorbance of the formazan product was 450 nm.

### 2.7 Adhesion and Inverted Centrifugation Studies

For DBCO-glasses, 5 mm-diameter glass coverslips functionalized with amino groups were acquired from Bioactive Surfaces SL and further functionalized with DBCO by incubating them overnight in 50 μg/cm^2^ DBCO-Sulfo-NHS with 0.45% DIPEA in 1× PBS. The next day, the coverslips were washed twice with 1× PBS containing 2% Triton and twice with 1× PBS only. They were then sterilized in an oven at 130 °C for 1 h. Nonfunctionalized coverslips were also sterilized to be used as adhesion controls and for laminin functionalization. Laminin-coated coverslips were prepared by covering 5 mm-diameter glass coverslips with 5 μg/mL laminin (Sigma, L2020), removing the excess solution, and allowing them to air-dry for 1 h. Sterile coverslips were glued to a 48-well plate using DOWSIL 738 Electrical Sealant (Dow). SCs were seeded onto the coverslips at a density of 3 × 10^4^ cells per well. For the click chemistry condition, SCs were previously incubated with Ac4ManNAz for 48 h. Cells were allowed to adhere for 30 min and 1 h, after which the coverslips were washed twice by repeatedly pipetting with 1× PBS. Cells that remained attached were fixed with 8% PFA and stained with 1 μg/mL DAPI for 20 min. Finally, fluorescence images were acquired at 2.5× magnification, and the number of cells on each coverslip was quantified.

For inverted centrifugation, SCs were seeded in a P60 at a density of 8 × 10^6^ cells and incubated with 40 μM Ac4ManNAz at 37 °C and 5% CO_2_ for 48 h. Then, cells were seeded over nonfunctionalized and DBCO-microfibers in a P24 multiwell plate and cultured for 24 h. After this time, the media was removed, and the cells were incubated in fresh media with 1 μM calcein (1923054, Invitrogen) for 15 min. Fluorescence images of 7 representative areas of the coverslip were taken at 10× magnification. Afterward, each coverslip was placed upside down in a 15 mL Falcon full of 1× PBS, and the coverslips were attached to the tube mouth with parafilm. Falcons were centrifuged for 5 min at 365*g*. After centrifugation, parafilm was removed, and coverslips were again placed in a clean P12 multiwell plate with fresh media in the same precentrifugation position, and fluorescence images in the same 7 locations were taken again. The mean value of the 7 fields was calculated for each coverslip, and one paired value per coverslip was used for statistical analysis. SCs attached to the microfibers were counted before and after the centrifugation, and the total number of cells was normalized by the number of microfibers in each picture.

### 2.8 Morphological Analysis

SCs were seeded in a P60 at a density of 8 × 10^6^ cells and incubated with 40 μM Ac4ManNAz at 37 °C and 5% CO_2_ for 48 h. Coverslips with nonfunctionalized and DBCO-microfibers were sterilized and placed in a P24 multiwell plate. Azide-SCs were raised with 1 mL of 0.05% trypsin and seeded at a density of 3 × 10^4^ cells per well and cultured for 10 days at 37 °C and 5% CO_2_. Glass coverslips without microfibers and disorganized microfibers were used as controls. Medium was replaced every 2 days. After this time, the medium was removed, and the cells were washed 3 times with 1× PBS (Phosphate Buffered Saline). Cells were fixed with 4% PFA for 20 min at room temperature. Once fixed, they were washed 3 times again with 1× PBS to remove traces of PFA.

Actin filament staining was performed by incubating the biohybrid scaffolds with Fluorescein (FITC)-labeled phalloidin (1:500, P5282, Merck) in 1× PBS with 2% Triton X-100, for 90 min at 4 °C. Finally, cell nuclei were stained, and samples were mounted in slides using Fluoroshield with DAPI (Sigma, F6057) and scanned using a Leica STELLARIS 8 confocal microscope. Confocal images were processed using ImageJ.

Alignment of SC nuclei relative to the microfiber axis was quantified using the directionality plugin in Fiji. For each image, the microfiber axis was defined as 0°, and the observed alignment fraction (*F*_observed_) was calculated as the fraction of the total directional signal falling within a ±10° interval around this axis. This value was normalized to the total directional signal distributed between −90° and +90°. To account for the fraction expected by random orientation alone, a chance-corrected alignment index was calculated using eq 2

2 $$\mathrm{alignment}\;\mathrm{index}=\frac{\left(F_{\mathrm{observed}}-F_{\mathrm{expected}}\;\mathrm{by}\;\mathrm{chance}\right)}{\left(1-F_{\mathrm{expected}}\;\mathrm{by}\;\mathrm{chance}\right)}$$

where *F*_expected_ by chance represents the fraction expected under a uniform random distribution of orientations and was calculated using eq 3

3 $$F_{\mathrm{expected}}\;\mathrm{by}\;\mathrm{ch}\mathrm{ance}=\frac{n\times w}{180^{{}^{\circ}}}$$

where *n* is the number of reference directions and *w* is the angular window size. In the aligned microfiber group, *n* = 1 and *w* = 20° (from −10° to +10°), resulting in *F*_expected_ by chance = 20/180 = 0.111. For the disorganized microfiber and no-microfiber groups, an arbitrary reference direction was used for comparison.

### 2.9 NSC-34 Coculture with Biohybrid Scaffolds: Neurite Alignment and Outgrowth, Overall Metabolic Activity, and NGF Levels

Azide-SCs were seeded in the different formats of microfibers at a density of 1 × 10^4^ cells per well in a P24 multiwell plate. Cells were cultured for 5 days at 37 °C and 5% CO_2_ in a commercial Schwann Cell Medium Kit previously described. At day 5, medium was removed and NSC-34 cells were seeded at a density of 1 × 10^4^ cells per well in Neurobasal-A Medium (10888–022, Gibco) supplemented with 5% FBS, 1% l-glutamine, 1% P/S, 1% B27 supplement (17504044, Gibco), 10 ng/mL Brain Derived Neurotrophic Factor (BDNF, 450–02, PeproTech), and 10 μg/mL Bovine Pituitary Extract (P1167, Sigma). Cells were cocultured at 37 °C and 5% CO_2_ for 7 and 15 days. NSC-34 seeded in aligned microfibers without SCs was taken as the negative control.

To calculate the length and orientation of NSC-34 neurites cultured with biohybrid scaffolds, an immunocytochemistry of the coculture was performed. To avoid breaking the axons, cells were fixed in a gradient at days 7 and 15. First, they were incubated in medium with 1% PFA for 2 min. Later, PFA was added until 2% concentration. Finally, cells were washed 2 times with 1x PBS for 5 min and incubated with 4% PFA for 10 min. Once fixed, cells were permeabilized in 1× PBS with 0.1% Triton for 10 min and blocked with 1× PBS with 2% Albumin fraction V (1.12018, Merck) for 1 h at room temperature. Cells were incubated with Mouse anti-βIII-tubulin antibody (1:1000, ab78078, Abcam) in 1× PBS with 0.1% Triton X-100 and 0.1% Albumin fraction V overnight at 4 °C. The next day, samples were washed and incubated with Fluorescein (FITC) anti-Mouse antibody (1:500, 115–095–003, Jackson ImmunoResearch) in 1× PBS with 0.1% Triton X-100 and 0.1% BSA for 4 h at room temperature. To visualize SCs’ cytoskeleton, cells were incubated with phalloidin–tetramethylrhodamine B isothiocyanate (Phalloidin-TRITC) (1:500, P1951, Merck) for 1 h at room temperature. Finally, cell nuclei were stained with 1 μg/mL DAPI for 20 min.

Representative images of different experimental groups were obtained using a Leica STELLARIS 8 confocal microscope. For neurite outgrowth and orientation analysis, fluorescence microscopy images were acquired using a Leica DMI3000 B inverted microscope with 10× magnification. The deviation angle of the neurites with respect to the microfiber direction was measured with the Directionality tool of ImageJ using the local gradient transformation method. The deviation degree of the neurites in each picture was calculated between 0 and 180° (these values correspond to a perfect alignment of the neurites with the microfibers). Using Microsoft Excel, the average amount of signal for each deviation angle was calculated in each experimental group and represented in the amount of signal *vs* deviation angle curves. Neurite length was measured using the NeuronJ plugin of ImageJ.

Overall metabolic activity of the coculture in the CC-linked BoB was evaluated using an AlamarBlueTM assay. After 7 days of coculture, the medium was removed from the wells, and 500 μL of fresh medium containing 10% alamarBlue reagent was added. As a negative control, the reagent mixture was added to a clean well without cells. Cocultures were incubated with the reagent at 37 °C and 5% CO_2_ for 3 h. After this time, the medium was transferred to a new multiwell plate, and cells were washed 3 times with 1× PBS for immunocytochemistry studies. alamarBlue absorbance was measured in a microplate reader at 570 and 600 nm. The percentage reduction of alamarBlue reagent was calculated using eq 4

4 $$\%reduction\;of\;alamarBlue\;reagent=\frac{\left(E_{oxi600}\times A_{570}\right)-\left(E_{oxi570}\times A_{600}\right)}{\left(E_{red570}\times C_{600}\right)-\left(E_{red600}\times C_{570}\right)}\times 100$$

where, *E*_oxi570_ is E of oxidized alamarBlue reagent at 570 nm = 80,586. *E*_oxi600_ is E of oxidized alamarBlue reagent at 600 nm = 117,216. *A*_570_ is the absorbance of test wells at 570 nm. *A*_600_ is the absorbance of test wells at 600 nm. *E*_red570_ is E of reduced alamarBlue reagent at 570 nm = 155,677. *E*_red600_ is E of reduced alamarBlue reagent at 600 nm = 14,652. *C*_570_ is the absorbance of the negative control well (media, AlamarBlue reagent, no cells) at 570 nm. C600 is the absorbance of the negative control well (media, AlamarBlue reagent, no cells) at 600 nm.

NGF levels in coculture supernatants were quantified. Supernatant medium (1 mL) was collected from all experimental groups at days 1, 7, and 15 and stored at −20 °C until analysis. For NGF quantification, a Rat β-NGF ELISA Kit (ERNGF, Invitrogen) was used following the manufacturer’s protocol. Because this assay specifically detects rat β-NGF, the measured signal was interpreted as the rat β-NGF fraction detected in the mixed coculture supernatant rather than as total NGF released by all cells in the system. 100 μL of samples and standards were added to the wells and incubated overnight with shaking at 4 °C. The next day, wells were washed 4 times with 1× PBS and incubated with 100 μL of biotin conjugate for 1 h with shaking at room temperature. Wells were washed 4 times again, and 100 μL of streptavidin–HRP was added and incubated for 45 min at room temperature with shaking. Finally, the wash step was repeated, and wells were incubated with 100 μL of TMB Substrate (substrate turns blue) for 30 min at room temperature in the dark with shaking. The reaction was terminated by adding 50 μL of Stop Solution to each well (the solution turns from blue to yellow). Absorbance was measured at 450 nm using an absorbance microplate reader (ELx800, BioTek Instruments). Because all experimental groups were maintained in the same supplemented coculture medium, these NGF measurements should be interpreted as a relative readout of the trophic milieu associated with each scaffold condition rather than as a direct measure of scaffold-specific neurotrophic signaling.

### 2.10 Statistical Analysis

Statistical analysis was performed using SPSS software (version 27.0; IBM, Armonk, NY, USA). Data are presented as mean ± standard error of the mean (SEM). Normality was assessed using the Shapiro–Wilk test for samples with *n* < 30. Comparisons between two independent groups were performed using Student’s *t-*test for normally distributed data and the Mann–Whitney U test for non-normally distributed data. Comparisons within the same sample were performed using a paired *t-*test. Comparisons among more than two groups were performed using one-way ANOVA followed by Bonferroni post hoc analysis for normally distributed data. For non-normally distributed data, statistical differences among groups were assessed using the Kruskal–Wallis test, and pairwise comparisons were performed using the Mann–Whitney U test. Differences were considered statistically significant at *p* < 0.05. Unless otherwise indicated, *n* refers to independent biological replicates (independent wells or coverslips prepared in separate experiments). For image-based assays, multiple ROIs or image fields acquired from the same biological replicate were averaged to obtain a single value per replicate and were not treated as independent replicates for statistical analysis.

All graphical representations were created using GraphPad Prism version 8.0.0 software for Windows (GraphPad Software, San Diego, California, USA), except the Amount of signal-Deviation angle curves, which were done using Microsoft Excel.

## 3 Results

### 3.1 Characterization and Conditioning of Silk Scaffolds for Click Chemistry

High-performance RSF microfibers were produced using the D3S technique. SF was obtained through degumming and dissolution of silk cocoons, and subsequently used for D3S spinning, as described in Section 2. SEM images were acquired to assess the morphology and surface topography (Figure 1A). The microfibers appear straight, uniform, and display a slightly rough surface. Thicker microfibers exhibited minor indentations along their length. We established an average microfiber diameter of 11.4 ± 4.1 μm (*n* = 21 individual microfibers). D3S enabled the controlled deposition of microfibers onto glass coverslips in two distinct architectural formats: aligned and disorganized. Both formats displayed comparable microfiber densities, indicating that the main structural difference between them was fiber orientation.

**Figure 1:**
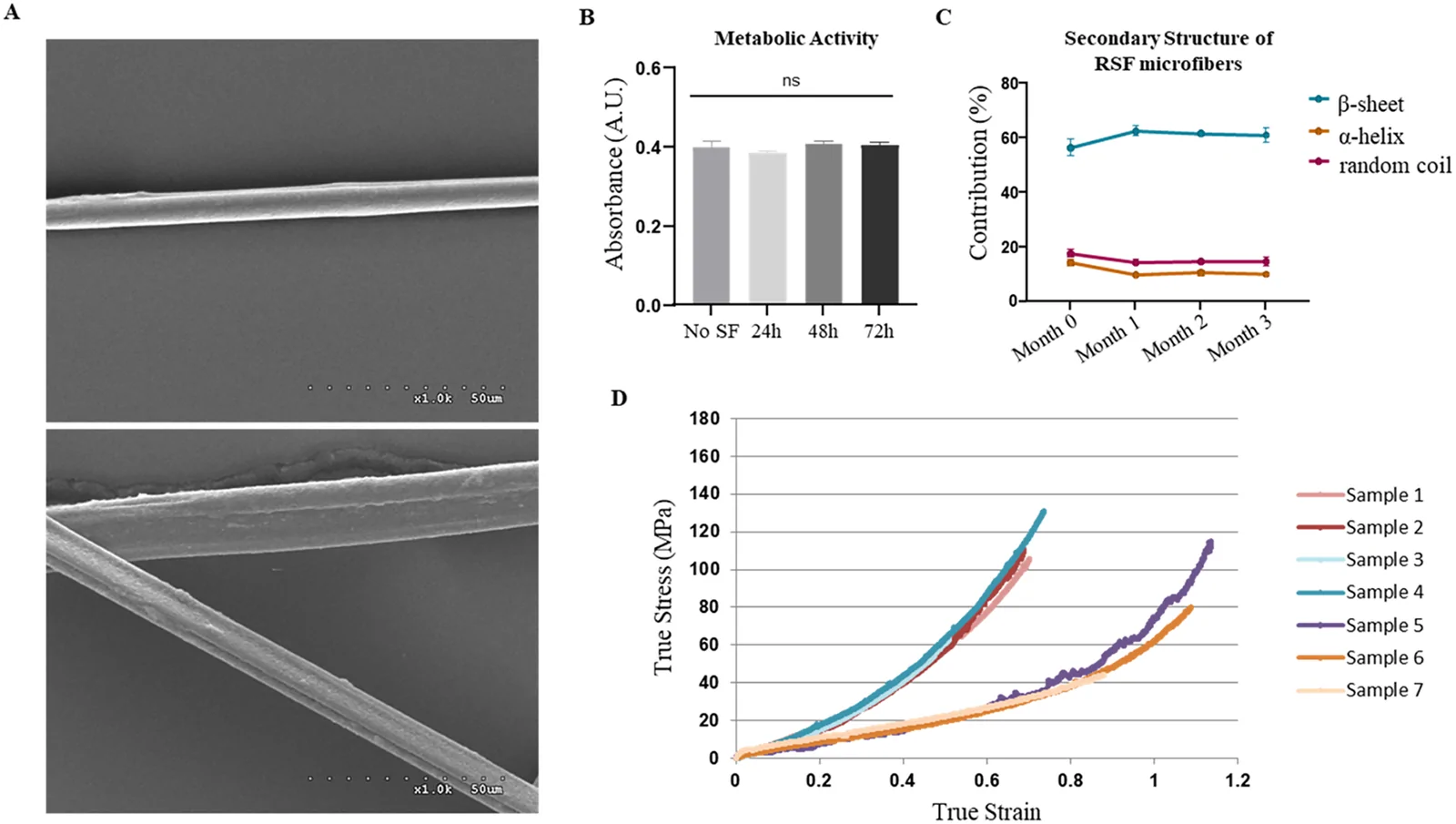
Topographical and mechanical characterization of RSF microfibers. (A) Scanning electron microscopy (SEM) images of RSF microfibers produced by D3S. Scale: 50 μm. (B) Metabolic activity of SCs cultured in media previously incubated with 2.5 mg RSF microfibers for 24, 48, and 72 h. No differences were found with respect to cells cultured with media not exposed to SF, indicating the noncytotoxicity of the biomaterial (*n* = 3 independent wells; ns indicates *p* > 0.05). (C) Changes in RSF microfiber crystallinity were evaluated by FTIR after incubation in distilled water at RT. A slight increase in the β-sheet content, accompanied by a decrease in α-helix and random coil structures, was observed after one month, indicating protein reorganization toward a more crystalline structure. No further changes in crystallinity were detected after 2 and 3 months of incubation, suggesting that the biomaterial remains structurally stable and does not undergo degradation or loss of its secondary structure (*n* = 3 independent replicates). (D) True stress–true strain curves of RSF microfibers (*n* = 7). Tensile tests were performed in water at room temperature.

The potential cytotoxicity of the D3S-produced RSF microfiber scaffolds was evaluated by using an XTT cell viability assay. Briefly, the biomaterial was incubated in a culture medium for 24, 48, or 72 h, and the resulting conditioned media were subsequently used to culture SCs for 24 h. As shown in Figure 1B, cell viability was not significantly different from that of SCs cultured in fresh medium that had not been exposed to the biomaterial, indicating that SF scaffolds do not release cytotoxic compounds.

To assess the stability and degradation behavior of the SF scaffolds under aqueous conditions, relevant to their intended application, RSF microfiber meshes were incubated in distilled water at RT and analyzed over time by ATR-FTIR spectroscopy. Changes in the secondary structure were evaluated by monitoring the relative content of the different conformational motifs. After one month of incubation, a slight increase in the β-sheet content was observed (from 56.5% to 62.6%), accompanied by a corresponding decrease in α-helical and random coil structures (from 14.2% to 9.6% for the first one and from 17.6% to 14.2% for the latter), suggesting a gradual reorganization of the protein toward a more crystalline conformation. No significant changes in secondary structure composition were detected after 2 or 3 months of incubation (Figure 1C).

We further characterized the mechanical properties of the high-performance RSF microfibers through tensile testing under hydrated conditions. The resulting true stress–true strain curves are shown in Figure 1D, while the measured diameter, maximum stress, elastic modulus, and work of fracture values for each sample (*n* = 7) are summarized in Table 2. The microfibers exhibited maximum stress values ranging from 45 to 131 MPa, with an average of 93 ± 32 MPa. The elastic modulus was relatively low, averaging 0.12 ± 0.05 GPa. Finally, the fibers displayed high toughness, with work of fracture values reaching up to 37 MJ/m^3^ and an average of 27.2 ± 8.7 MJ/m^3^.

**Table 2:** Tensile Test Results of High-Performance RSF Microfibers Produced by D3S^a^

sample	diameter (μm)	maximum stress (MPa)	elastic modulus (GPa)	work of fracture (MJ/m^3^)
1	9.9	107	0.15	29.2
2	13.0	111	0.16	27.1
3	10.5	60	0.12	13.1
4	9.6	131	0.17	35.2
5	10.0	115	0.09	37.6
6	11.0	80	0.07	29.6
7	11.8	45	0.05	18.7
Mean ± SD	10.8 ± 1.2	93 ± 32	0.12 ± 0.05	27.2 ± 8.7

a Maximum stress, elastic modulus, and work of fracture were determined from the true stress–true strain curves for each microfiber (*n* = 7).

To enable covalent cell attachment *via* click chemistry, the microfiber surface was functionalized with dibenzocyclooctyne-*N*-hydroxysuccinimidyl ester (DBCO-NHS). We functionalized 4 mg of SF microfibers with a concentration of 320 μg/mL overnight and washed several times to remove the remnants. To verify the successful functionalization, DBCO-modified microfibers were incubated with a fluorescein (FAM) azide dye. As shown in Figure 2A, DBCO-functionalized microfibers exhibited strong green fluorescence, whereas nonfunctionalized microfibers (highlighted by yellow dashed lines) showed negligible labeling. Quantification of fluorescence intensity revealed statistically significant differences between the two groups (*p* < 0.0001, Figure 2B), corroborating the visual observations. Functionalization with DBCO was further assessed by UV–vis spectroscopy. Both DBCO-functionalized and nonfunctionalized microfibers were dissolved in LiBr to obtain homogeneous solutions, and the absorbance was measured at 310 nm, corresponding to the maximum absorption peak of DBCO. A significant increase in absorbance was observed for the DBCO-functionalized microfibers compared to the nonfunctionalized controls (*p* = 0.0044), indicating the successful attachment of DBCO to the microfiber surface. Additionally, the concentration of DBCO groups covalently bound to the microfibers was quantified by interpolating the absorbance values at 310 nm using a DBCO-NHS standard curve. We determined a concentration of 94.7 ± 25.8 μg/mL DBCO linked to 4 mg of SF microfibers or a DBCO density of 23.4 ± 6.4 nmol DBCO/mg SF (mean ± SD) calculated using eq 1.

**Figure 2:**
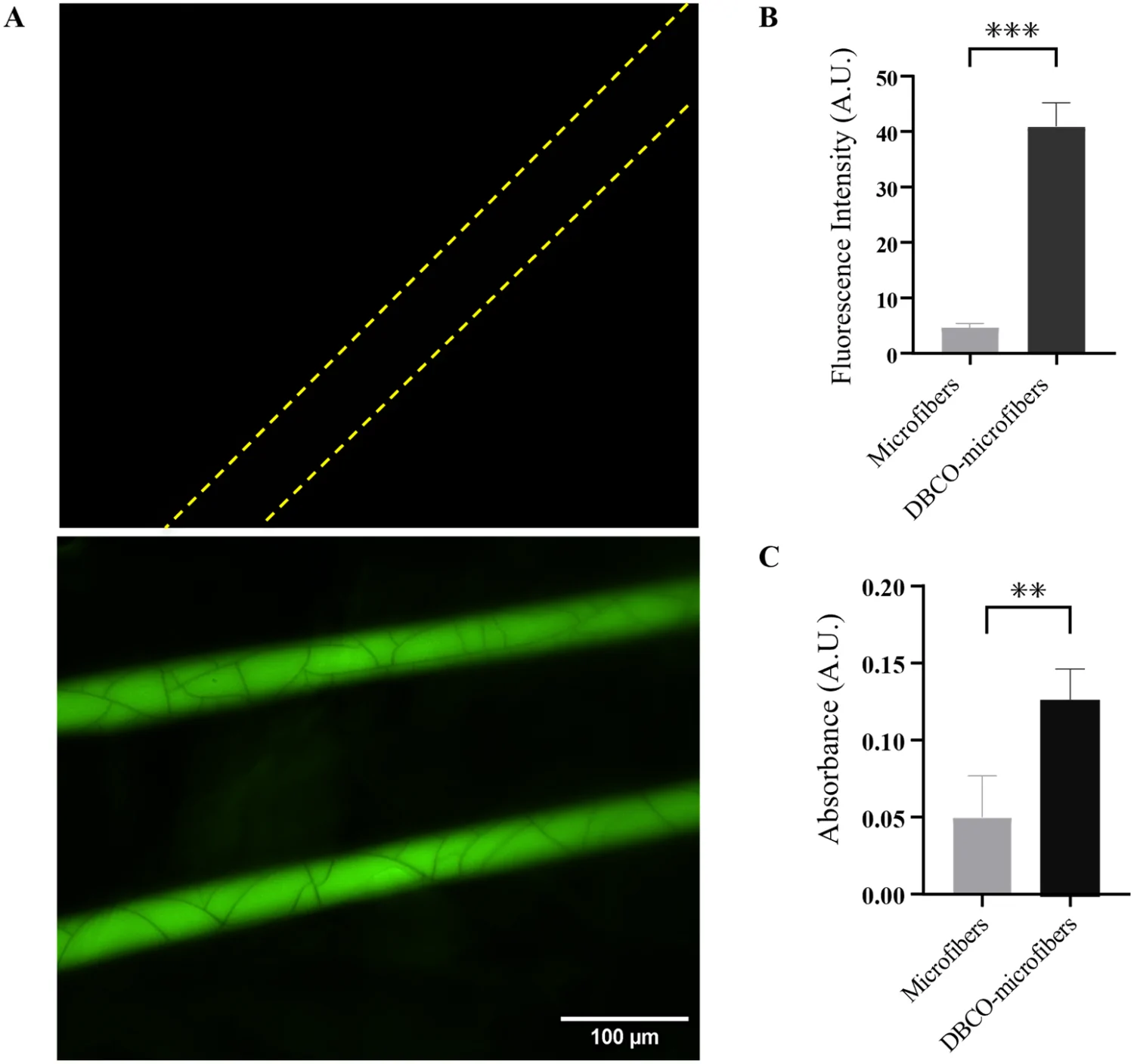
Scaffold conditioning for click chemistry: functionalization with DBCO. (A) Fluorescence images of DBCO-functionalized microfibers labeled with FAM azide dye. After functionalization with DBCO, incubation with FAM azide was performed. Positive labeling of DBCO-microfibers (green) in contrast with nonfunctionalized microfibers (indicated by yellow dashed lines) indicates the successful conditioning of the scaffold. Scale bar: 100 μm. (B) Fluorescence intensity of nonfunctionalized (*n* = 4 individual microfibers) and DBCO-functionalized microfibers (*n* = 11 individual microfibers) shows significant differences between groups, supporting successful scaffold functionalization. Fluorescence intensity was measured in ROIs of constant area placed on the microfiber surface, and ROI values from the same microfiber were averaged to obtain one value per microfiber (*** indicates *p* < 0.001). (C) Absorbance of 4 mg SF microfibers and DBCO-microfibers (*n* = 4) dissolved in LiBr and measured at 310 nm. The significant increase in absorbance (*p* = 0.0044) corroborates the successful incorporation of DBCO over the microfiber’s surface.

### 3.2 Cell Conditioning for Click Chemistry

SCs were prepared for covalent attachment using azide-sugar metabolic glycoengineering. Due to the limited literature on the application of this technique in SCs, the protocol was optimized in this study. N-Azidoacetylmannosamine-tetraacylated (Ac4ManNAz) was employed to introduce azide groups on the cell surface. Two concentrations of Ac4ManNAz (20 μM and 40 μM) and three incubation times (24, 48, and 72 h) were tested. To assess the effectiveness of the functionalization, cells were incubated with a Cyanine3–DBCO dye (red), and fluorescence microscopy images were acquired (Figure 3A) to quantify fluorescence intensity (Figure 3B). No significant differences were observed with 20 μM Ac4ManNAz throughout the study period. In contrast, significant increases in surface azide expression were detected with 40 μM Ac4ManNAz after 24 h (*p* = 0.018) and 48 h (*p* = 0.001) compared to nonfunctionalized SCs. These differences disappeared after 72 h.

**Figure 3:**
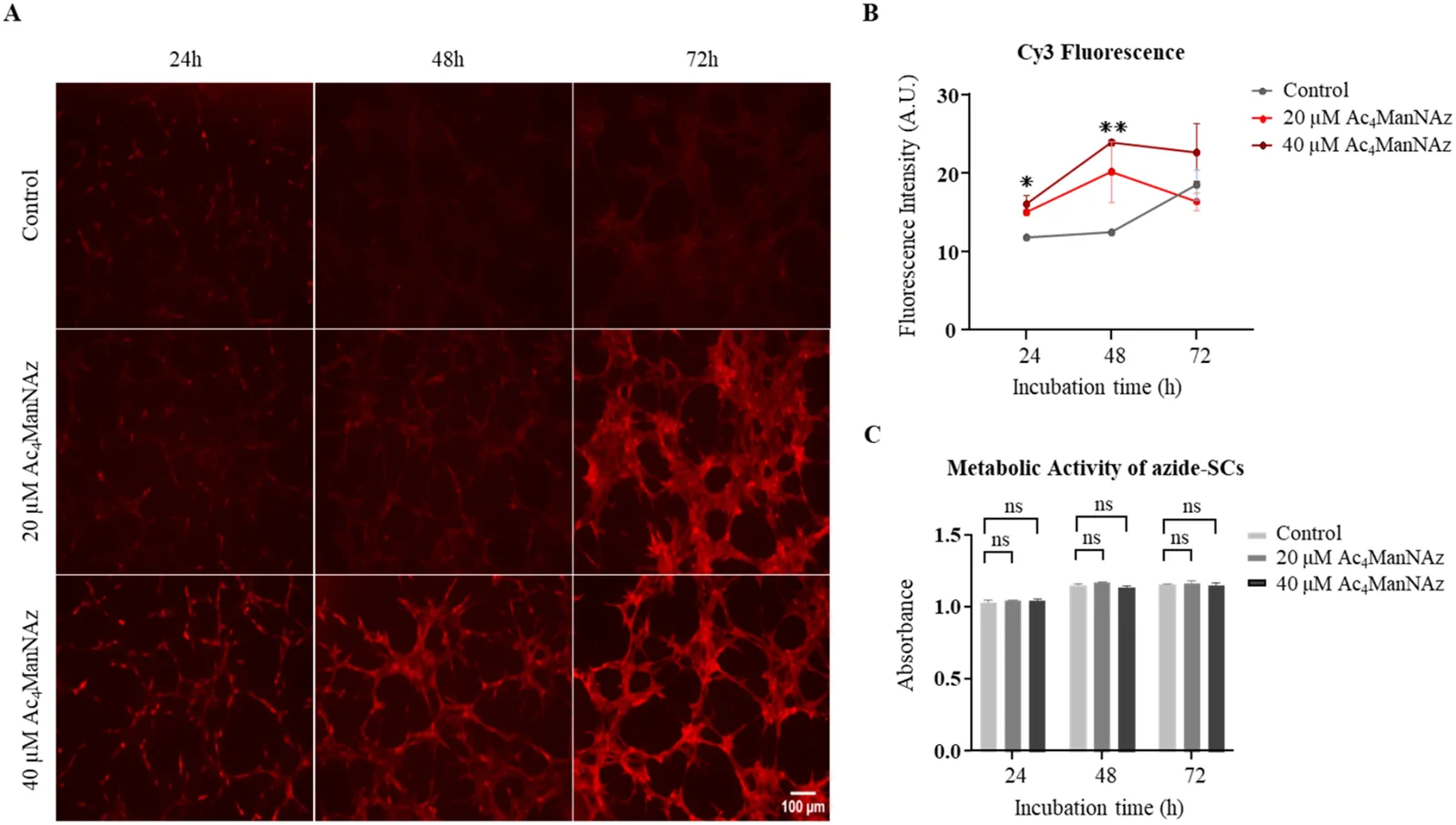
Cells conditioning for click chemistry: azide-sugar functionalization of SCs. (A) Fluorescence microscopy images of azide-SCs, labeled with Cyanine 3-DBCO dye. SCs were first incubated with 20 μM and 40 μM of Ac_4_ManNAz for 24 h, 48 h, and 72 h, and then labeled with a Cyanine 3-DBCO dye (red) to verify the presence of azide groups in the cell surface. Scale: 100 μm. (B) Quantification of fluorescence intensity shows significant differences after incubation with 40 μM Ac_4_ManNAz for 24 and 48 h but not with 20 μM Ac_4_ManNAz (*n* = 3 independent wells). (C) Metabolic activity of SCs incubated with 20 μM and 40 μM Ac_4_ManNAz for 24 h, 48 h, and 72 h shows no differences with respect to controls, indicating that Ac_4_ManNAz is not cytotoxic for SCs (*n* = 4 independent wells; ns indicates *p* > 0.05, * indicates *p* < 0.05, and ** indicates *p* < 0.01).

The potential cytotoxicity of Ac4ManNAz in SCs was evaluated using an XTT cell proliferation assay after incubation with Ac4ManNAz. As shown in Figure 3C, no differences were observed between SCs cultured with fresh media and SCs incubated with 20 μM and 40 μM Ac4ManNAz for 24, 48, and 72 h, confirming that Ac4ManNAz is not cytotoxic for SCs.

### 3.3 Click Chemistry Reinforces Cell Attachment to the Biomaterial

We verified that the click chemistry reaction takes place and validated that covalent bonding enables stronger adhesion compared with noncovalent interactions. SCs were seeded onto nonfunctionalized, laminin-coated, and DBCO-functionalized coverslips. For the click chemistry condition, we used azide-SCs previously functionalized. Cells were allowed to adhere for either 30 min or 1 h, after which the coverslips were washed twice with 1× PBS by repeatedly pipetting. The remaining cells were then fixed and stained with DAPI. Fluorescence imaging (Figure 4A) shows a high number of adherent cells in the click chemistry group, whereas very few cells are observed on nonfunctionalized or laminin-coated coverslips at both times. Quantification of cell numbers normalized to the coverslip surface corroborates these observations, revealing significant differences between click chemistry and untreated surfaces after 30 min of adhesion (*p* < 0.0001) and 1 h of adhesion (*p* < 0.0001) as well as between click chemistry and laminin-coated surfaces after 30 min (*p* < 0.0001) and 1 h (*p* < 0.0001).

**Figure 4:**
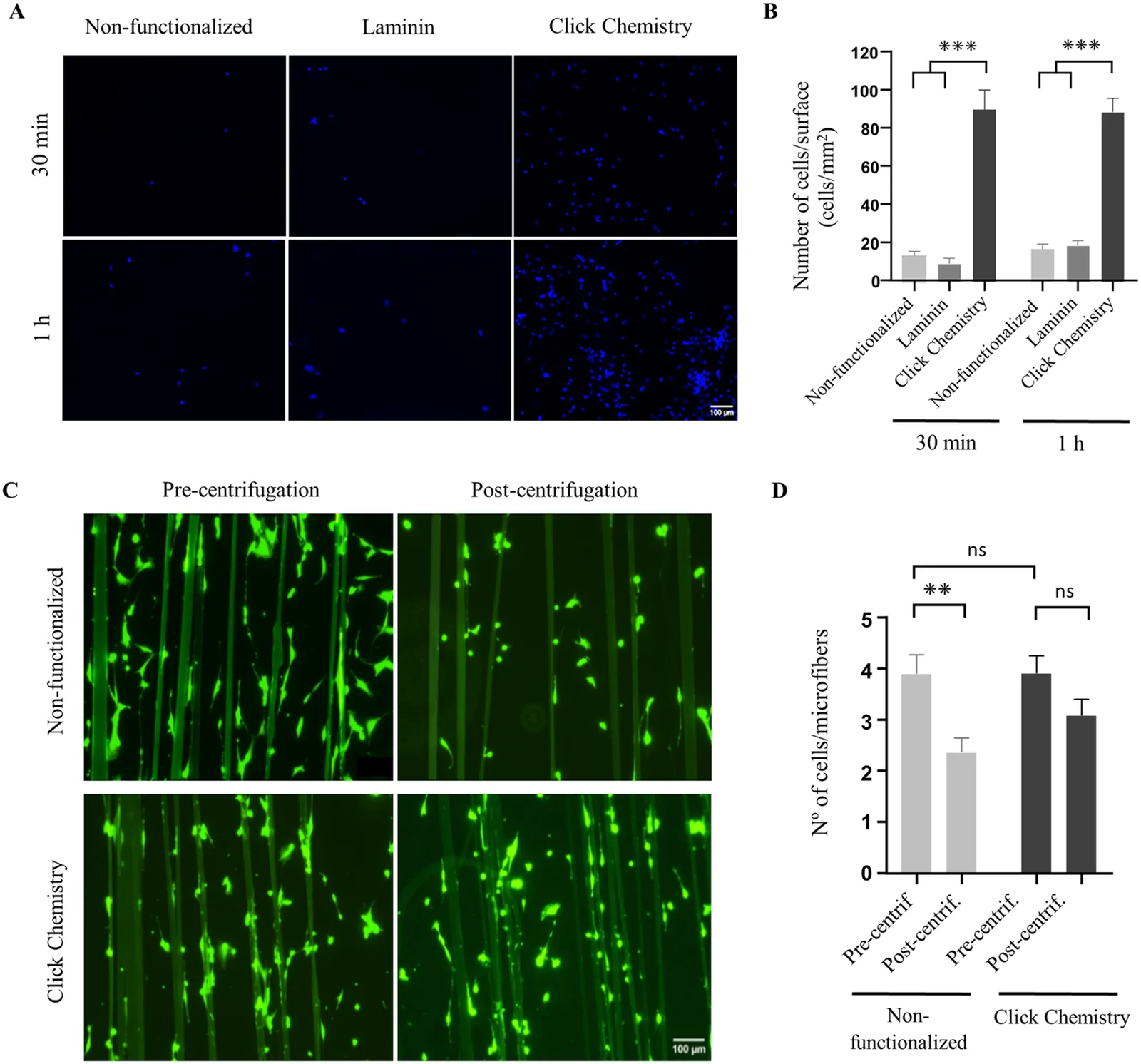
Cell-scaffold attachment by click chemistry. (A) DAPI staining of SCs and azide-SCs seeded over nonfunctionalized, laminin-coated, and DBCO-functionalized coverslips. The cells were allowed to adhere for 30 min and 1 h, after which the coverslips were washed twice. Scale bar: 100 μm. (B) Quantification of the number of cells normalized by the coverslip surface in the nonfunctionalized, laminin-coated, and DBCO coverslips (*n* = 4 independent coverslips). (C) Fluorescence images of azide-SCs cultured over nonfunctionalized and DBCO-functionalized microfibers (Click Chemistry) after 24 h and stained with calcein. Representative pictures were acquired before and after inverted centrifugation. Scale bar: 100 μm. (D) Quantification of the number of cells normalized by the number of microfibers in each coverslip (*n* = 11 independent coverslips) pre- and post-inverted centrifugation. Statistical analysis shows a significant decrease in the number of SCs after centrifugation when no functionalization is applied. In contrast, there are no significant differences in the number of SCs linked by click chemistry, indicating stronger attachment between cells and biomaterial (ns indicates *p* > 0.05 and ** indicates *p* < 0.01).

We also evaluated the covalent attachment by click chemistry between cells and microfibers by using an inverted centrifugation assay. Azide-SCs were cultured on nonfunctionalized and DBCO-functionalized microfibers for 24 h. Cells were then stained with calcein, and representative images were captured before and after the inverted centrifugation (Figure 4C). The number of cells per microfiber was quantified for each replicate, and pre- and postcentrifugation values were statistically compared within each group. As shown in Figure 4D, there were no differences between nonfunctionalized microfibers and those functionalized with click chemistry before centrifugation, indicating that click chemistry functionalization does not increase the number of cells attached to the biomaterial. However, the number of adherent cells decreased significantly after centrifugation on nonfunctionalized microfibers (*p* = 0.004). In contrast, the click chemistry group exhibited only a minor reduction in cell number, with no significant differences between pre- and postcentrifugation conditions (*p* = 0.094).

### 3.4 Click Chemistry-Linked Biohybrid Scaffolds Promote Directed Neurite Outgrowth *In Vitro*

We aimed to validate that covalent adhesion does not interfere with the functional properties of the biohybrid scaffolds. As a proof of concept, we created BoB-like constructs by covalently linking SCs to SF microfibers through click chemistry and evaluated their biological performance *in vitro*. First, we verified the ability of SCs to elongate and align with the anisotropic scaffold. SCs were metabolically labeled with 40 μM Ac_4_ManNAz for 48 h, as this condition was established as the optimal conditioning protocol based on prior optimization experiments. Azide-SCs were then seeded onto DBCO-microfibers, while nonazide SCs were seeded over nonfunctionalized microfibers. We used two isotropic controls: cells seeded over disorganized microfibers and glass coverslips without microfibers. Cells were cultured for 10 days and subsequently stained with phalloidin and DAPI. As shown in Figure 5A, SCs cultured on disorganized or without microfibers showed a random growth pattern, extending in multiple directions. In contrast, SCs grown on aligned microfibers elongated along the microfiber axis, forming longitudinal cell arrays reminiscent of *in vivo* BoB’s architecture. Notably, in the nonfunctionalized aligned and disorganized groups, SCs were often observed bridging the spaces between adjacent microfibers. In contrast, with click chemistry adhesion, cells appeared to remain more tightly associated with the microfiber surface. We quantified SCs alignment by measuring the orientation of SCs nuclei relative to the microfiber axis. For each image, the microfiber axis was defined as 0°, and the observed alignment fraction (*F*_observed_) was calculated as the fraction of the total directional signal falling within a ±10° interval around this axis. This value was normalized to the total directional signal distributed between −90° and +90°. To account for the fraction expected by random orientation alone, a chance-corrected alignment index was calculated using eqs 2 and 3. For the disorganized microfiber and no microfiber groups, an arbitrary reference direction was used for comparison. We found that the alignment index of SC nuclei was significantly higher on click chemistry-aligned microfibers than on empty coverslips (*p* = 0.0286) and disorganized microfibers (*p* = 0.0286). Alignment was also significantly higher on nonfunctionalized aligned microfibers than on anisotropic controls (*p* = 0.0286). These results indicate that click-mediated attachment did not impair SC elongation or directional organization on either aligned or disorganized microfibers. Overall, this supports the feasibility of covalent coupling while preserving the ability to generate spatially organized, directional biohybrid constructs.

**Figure 5:**
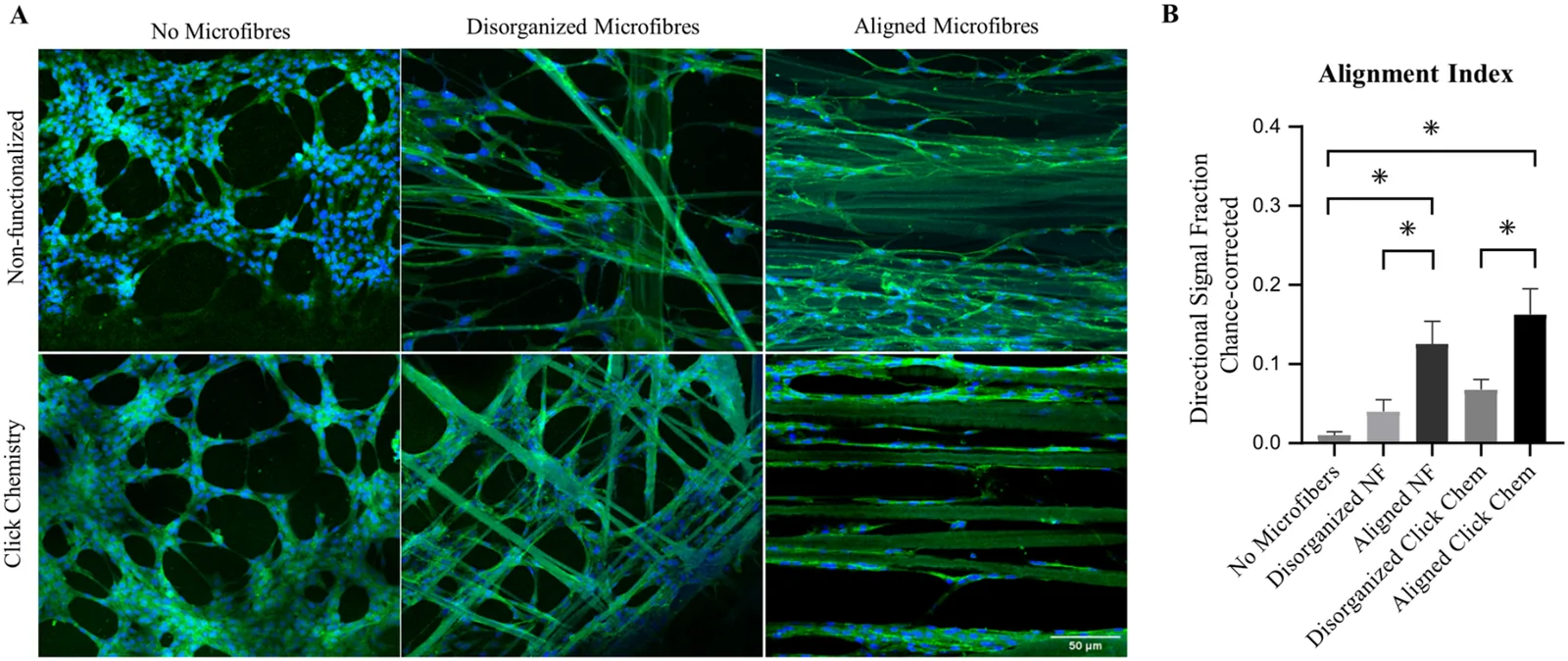
SCs linked by click chemistry to the scaffold develop BoB *in vitro*. (A) Fluorescence images of azide-SCs cultured over nonfunctionalized and DBCO-functionalized microfibers (Click Chemistry) after 10 days. Aligned microfibers induce SCs elongation, developing longitudinal rows that mimic BoB architecture, unlike empty coverslips or disorganized microfibers that yield network-like cell patterns. Click chemistry allows cell alignment with microfibers, showing no detrimental effect on SCs elongation or spatial organization for up to 10 days. The cytoskeleton was stained with phalloidin (green), and the cell nuclei were stained with DAPI (blue). Scale bar: 50 μm. (B) SC nuclei alignment index in no microfiber, disorganized microfiber, and aligned microfiber groups, with or without click-chemistry functionalization (*n* = 4 independent coverslips). The alignment index was calculated as the fraction of total directional signal within ± 10° of the microfiber axis (0°). A chance correction was applied by subtracting the expected fraction of directional signal (calculated based on random distribution) from the observed fraction (* indicates *p* < 0.05).

Once created, we assessed whether our click chemistry-linked BoB (CC-linked BoB) could support directed neurite outgrowth without loss of biological functionality. For this purpose, we cultured a commercial line of motor neurons (NSC-34) in contact with CC-linked BoB for 7 and 15 days and analyzed neurite length and orientation. As our goal was not to determine the independent effect of click chemistry on neural regeneration, but to verify that it does not impair the functionality of the biohybrid construct, we included two negative controls designed to reduce neural guidance and neurite outgrowth: disorganized microfibers with SCs (loss-of-anisotropy control) and aligned microfibers without SCs (loss-of-cellular-support control). Figure 6A shows that neurites grew preferentially along the direction of aligned microfibers, resulting in high signal intensity at deviation angles of ± 10° (Figure 7A). This alignment profile was comparable to that observed in oriented microfibers with noncellular support, indicating that scaffold anisotropy was the main driver of directional guidance. In contrast, disorganized microfibers promoted neurite growth in multiple directions, leading to a broader angular distribution (Figure 7A). Although alignment was largely determined by scaffold architecture, the presence of SCs in the aligned biohybrid scaffold enhanced neurite extension. As shown in Figure 7B, neurites grown on CC-linked BoB were significantly longer than those in the disorganized format after 1 week in culture (*p* = 0.002). Moreover, CC-linked BoB supported significantly longer neurites than aligned microfibers without SCs after one (*p* = 0.002) and 2 weeks (*p* = 0.029). Together, these results indicate that the anisotropic scaffold mainly provides directional cues, whereas SC incorporation contributes more strongly to neurite outgrowth.

**Figure 6:**
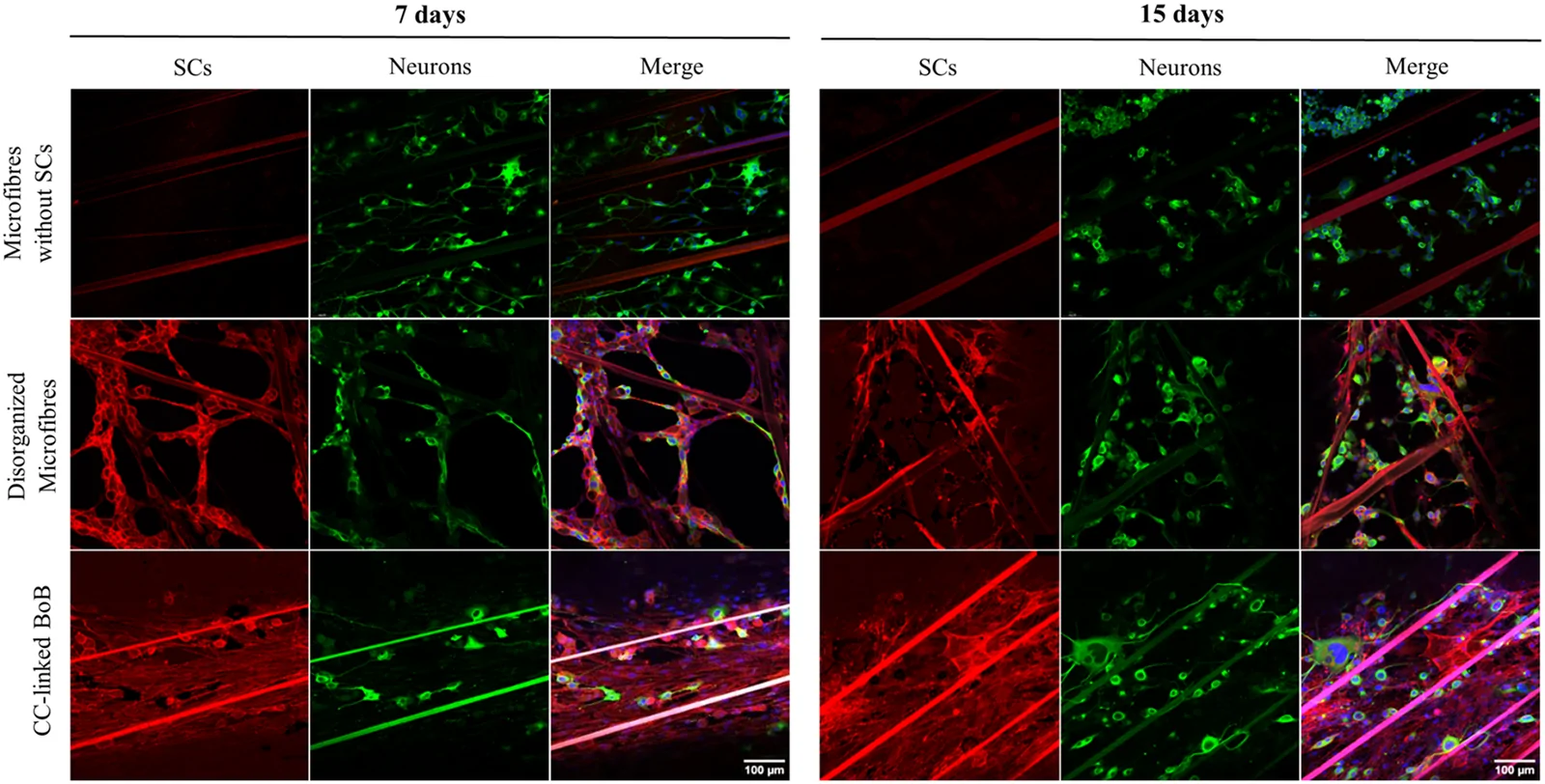
CC-linked BoB supports directed neurite outgrowth *in vitro*. Confocal images of NSC-34 neurons cultured on different microfiber scaffold formats after 7 and 15 days. In aligned microfibers without SCs (first row), neurites aligned with the microfiber axis but showed reduced extension. In disorganized biohybrid scaffolds containing SCs (second row), neurites extended in multiple directions, reflecting the loss of anisotropic guidance. In contrast, CC-linked BoB based on aligned microfibers and SCs (third row) supported both directional neurite growth and greater neurite extension. Neurons are shown in green (β-III-tubulin), SCs in red (phalloidin), and nuclei in blue (DAPI). Scale bar: 100 μm.

**Figure 7:**
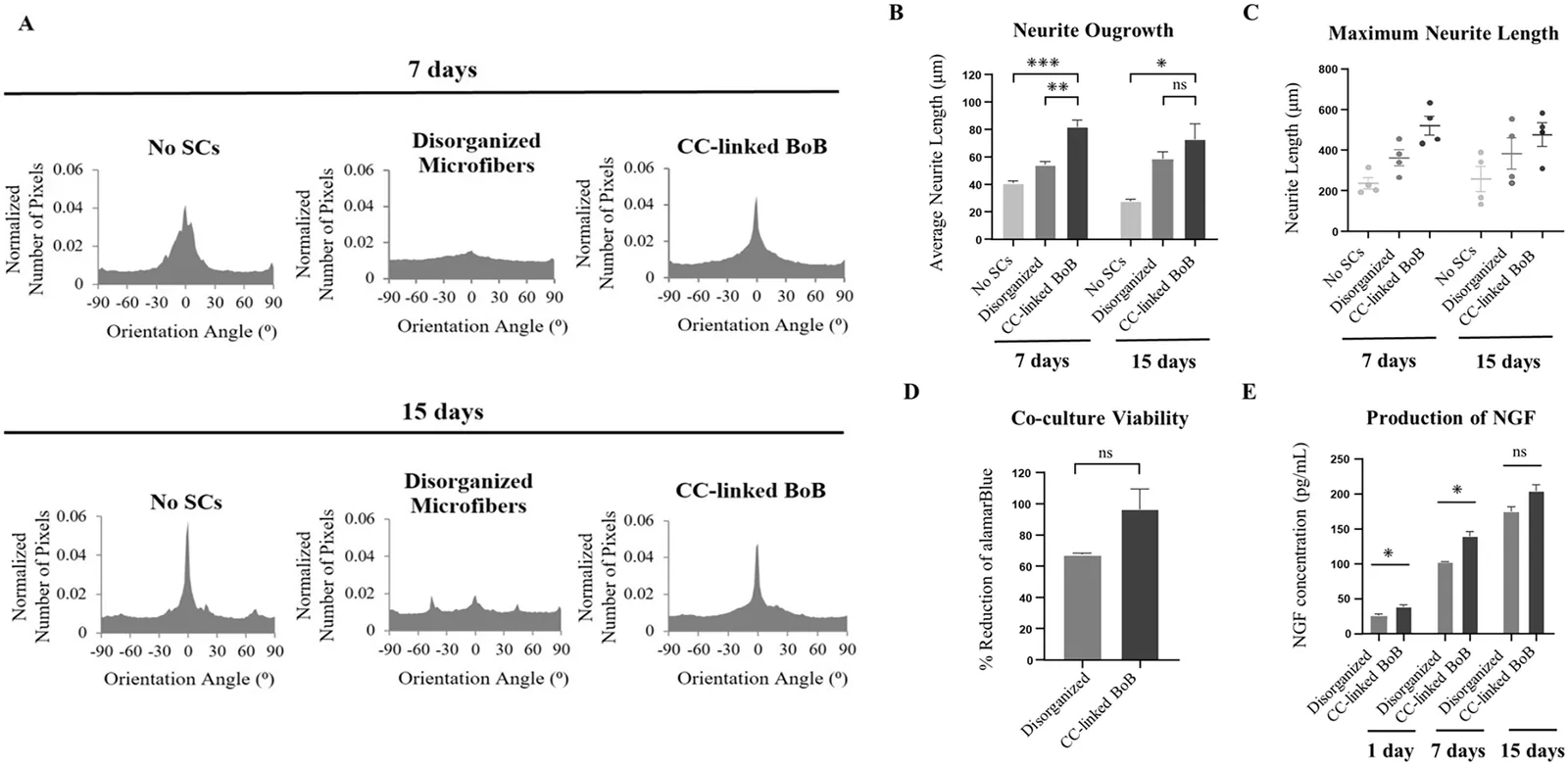
CC-linked BoB supports directed neurite outgrowth *in vitro*. (A) Graphical representation of β-III-tubulin fluorescence *versus* deviation angle relative to microfiber direction (0°). In both CC-linked BoB and aligned microfibers without SCs, neurites showed preferential alignment along the microfiber axis after 7 and 15 days, indicating that scaffold anisotropy is the main determinant of directional guidance. In contrast, neurites cultured on disorganized microfibers displayed a broader angular distribution (*n* = 4 independent coculture replicates). (B) Quantification of average neurite length shows significantly greater neurite outgrowth in CC-linked BoB than in disorganized microfibers after 7 days, and then in aligned microfibers without SCs after 7 and 15 days (*n* = 4 independent coculture replicates), indicating that SC incorporation enhances neurite extension. (C) Maximum neurite lengths of each replicate are shown as descriptive data. (D) AlamarBlue assay shows comparable overall coculture metabolic activity after 1 week in CC-linked BoB and disorganized biohybrid scaffolds (*n* = 3 independent coculture replicates). (E) Quantification of rat β-NGF in coculture supernatants shows significantly higher detected levels in CC-linked BoB after 1 and 7 days, with a similar nonsignificant trend after 15 days (*n* = 3 independent coculture replicates from independent experiments; ns indicates *p* > 0.05, * indicates *p* < 0.05, ** indicates *p* < 0.01, and *** indicates *p* < 0.001).

Additionally, we studied the overall metabolic activity of the coculture with an AlamarBlue assay using eq 4. As shown in Figure 7D, after 7 days, CC-linked BoB showed metabolic activity comparable to that of disorganized biohybrid scaffolds, with no significant differences between the two conditions (p = 0.084). We also quantified NGF levels in supernatants from aligned and disorganized biohybrid scaffolds cocultured with NSC-34 after 1, 7, and 15 days. As shown in Figure 7E, the rat β-NGF signal detected in CC-linked BoB was significantly higher after 1 day of coculture (*p* = 0.022) and remained significantly higher after 1 week (*p* = 0.005). A similar trend was observed after 2 weeks, although differences did not reach statistical significance at that time point (*p* = 0.063). Since all groups were maintained in the same supplemented coculture medium, these data were interpreted as a relative readout of the trophic milieu associated with each scaffold condition. Together, these results indicate that click-mediated assembly does not impair the biological activity of the biohybrid scaffolds.

## 4 Discussion

In the last few decades, tissue engineering has explored the incorporation of cells into artificial constructs to generate chemical signals that enhance the regenerative efficacy of implanted scaffolds. The most common strategies involve molecules that mimic the natural ECM of the target tissue, providing reversible and nonspecific adhesion that enables cell migration within the scaffold.^8,9^ However, noncovalent interactions are relatively weak and can be influenced by the tissue environment after implantation, potentially compromising scaffold cellularization and overall effectiveness. In contrast, biorthogonal click chemistry offers a promising solution, as it enables the formation of covalent bonds between specific molecules under physiological conditions. In the present study, we propose the application of click chemistry—specifically SPAAC ligation—in the field of tissue engineering to improve adhesion between cells and biomaterials. As a proof of concept, we engineered artificial BoB by combining SF microfibers and SCs and evaluated their biological performance *in vitro*.

First, we produced high-performance RSF microfibers using D3S. This technique allows us to produce SF fibers with micrometer diameters while enabling their deposition with controlled directionality. This way, we produce aligned microfibers that mimic BoB architecture and compare them with a disorganized organization. SEM images of microfibers revealed a straight and uniform morphology and a slightly rough surface, which may be advantageous since irregular topographies are known to promote cell adhesion. We studied the potential cytotoxicity of the RSF microfibers produced by the D3S in SCs. Although silk sericin is removed during the degumming process prior to spinning, trace amounts of reagents used during D3S production (ethanol, acetic acid, and lithium bromide) could potentially remain after dialysis and sterilization and may contribute to the cytotoxic effects. Favorably, no difference in cell metabolic activity was found after 24, 48, or 72 h of exposure to the conditioned medium in comparison with fresh media, which confirms the suitability of the SF scaffolds as biocompatible substrates for SCs.

We also assessed the stability and degradation behavior of the SF scaffolds under aqueous conditions using ATR-FTIR spectroscopy. Only a slight increase in β-sheet content, accompanied by a corresponding decrease in α-helical and random-coil structures, was observed, suggesting a gradual reorganization of the protein toward a more crystalline conformation. This transition may be attributed to interactions between the protein chains and water molecules. No significant changes in secondary structure were detected after 2 or 3 months of incubation, indicating that the RSF microfibers largely preserve their structural integrity over time. These results suggest that the scaffolds do not undergo substantial degradation or loss of crystallinity under the conditions tested. Additionally, we characterized the mechanical properties of the biomaterial by tensile testing. The microfibers exhibited maximum stresses of up to 131 MPa, highlighting the strength of the biomaterial. The elastic modulus of the RSF microfibers was relatively low (∼0.12 GPa), a range commonly observed in structural biopolymers such as silk or collagen. Additionally, the work of fracture reached values of up to 37 MJ/m^3^, reflecting their high toughness and overall quality. Collectively, these mechanical properties highlight the adequacy of the biomaterial in terms of strength and flexibility, which is encouraging from a translational perspective, although additional studies are needed to assess surgical handling and *in vivo* performance.

Once produced, the biomaterials were prepared for click chemistry functionalization. We introduced DBCO groups onto the microfiber surface using a DBCO–NHS ester, whose NHS group exhibits high reactivity toward amino groups in SF proteins. The success of the functionalization was verified using two complementary methods. First, we labeled the samples with a fluorescein–azide probe and quantified fluorescence intensity, observing significantly higher binding in DBCO-functionalized microfibers compared to nonfunctionalized ones. Second, we verified the presence of DBCO groups in the microfibers by spectrophotometry, as DBCO exhibits a characteristic absorption peak at 310 nm that is absent in SF. As we expected, a significant increase in absorbance was observed for the DBCO-functionalized microfibers, confirming the presence of DBCO on the microfiber surface at a concentration of 23.4 ± 6.4 nmol DBCO/mg SF. The variability observed was interpreted as a consequence of the intrinsic heterogeneity of the D3S microfiber production process. Although all replicates were prepared using the same mass of SF, D3S fabrication yielded microfibers with diameters of 11.4 ± 4.1 μm (*n* = 21), corresponding to a relative variation of approximately 36% (Coefficient of Variation, CV = Mean/SD × 100). Consequently, microfibers with the same mass may exhibit different total surface areas and, therefore, different numbers of reactive amino groups available for DBCO conjugation. For instance, assuming a cylindrical geometry and a constant microfiber length of 1 μm, the lateral surface area of the measured microfibers is ∼35.8 ± 13 μm^2^. Based on the reported lysine content of SF (≈0.3 mol %),^42,43^ a theoretical density on the order of 10^4^ amino groups/μm^2^ can be estimated, with an average of 35.8 ± 13 × 10^4^ amino groups (CV = 36%) in the microfiber’s surface. Therefore, variations in microfiber dimensions alone could result in substantial differences in the number of available reactive sites and, consequently, in the amount of conjugated DBCO.

On the other hand, we modified the surface of SCs through metabolic glycoengineering to enable click chemistry attachment. By incubating the cells with a synthetic azido-sugar (Ac4ManNAz), we introduced azide groups into the glycoproteins and glycolipids of the cell membrane. Different concentrations and incubation times were tested to identify the optimal conditions for SC functionalization while maintaining cell viability. We checked the presence of azide groups in the cells by labeling with a Cyanine 3-DBCO dye and quantifying the fluorescence intensity. We found that SCs began to show significantly higher azide expression on the cell surface after 24 h using 40 μM of Ac4ManNAz, reaching the higher differences after 48 h. However, no significant differences were found after 72 h in comparison with nonfunctionalized SCs, probably caused by cell membrane renewal and the elimination of glycoproteins labeled with azide. The viability assay showed no cytotoxic effects at the used concentrations. Therefore, we establish 40 μM of Ac4ManNAz and 48 h as the optimal conditions for SCs functionalization with azide.

After conditioning the microfibers and the cells for click chemistry, we next verified that the reaction occurred and that the resulting covalent bonds effectively enhanced adhesion strength beyond that achieved through noncovalent interactions. To this end, we compared SC adhesion to nonfunctionalized, laminin-coated, and DBCO-functionalized coverslips after 30 min and 1 h. Laminin was selected as a control for noncovalent adhesion because, along with polylysine, it is one of the most used ECM-derived molecules to promote adhesion in SCs. The number of cells remaining on the coverslips after washing was very low on the untreated and laminin-coated surfaces, whereas significantly more cells remained attached under the click-chemistry conditions. This corroborates that covalent chemistry provides a stronger adhesion compared with noncovalent methods. We further assessed the efficacy of click chemistry in promoting SC adhesion to SF microfibers, specifically using a centrifugation-based detachment assay. Centrifugation markedly reduced the number of cells attached to nonfunctionalized microfibers. In contrast, DBCO-functionalized microfibers exhibited only a small, nonsignificant decrease in cell number, consistent with covalent cell-scaffold linkage.

As a proof of concept, we used click chemistry to covalently link SCs to SF microfibers and evaluated whether a stable and biologically functional BoB-like scaffold could be assembled *in vitro*. Because our aim was not to determine the independent effect of click chemistry on neural regeneration but rather to confirm that covalent assembly does not compromise scaffold function, we included two negative controls expected to reduce neurite guidance or outgrowth: disorganized microfibers seeded with SCs and aligned microfibers without SCs. This comparison helps distinguish the contribution of scaffold architecture from that of cellular support, although it does not isolate the specific effect of the click chemistry itself. Our observations indicate that the anisotropic microfiber architecture provides the main physical cue for neurite alignment, whereas the incorporation of SCs contributes more directly to neurite extension and biological support. Within this framework, the role of click chemistry is to enable stable cell-scaffold assembly while preserving the SC organization, scaffold integrity, and biological function over time. Indeed, SCs covalently linked to aligned microfibers formed elongated longitudinal rows reminiscent of native BoB and maintained the construct for at least 10 days. When cocultured with neurons, these biohybrid scaffolds supported directed neurite outgrowth and longer neurites than the negative controls throughout 2 weeks, while preserving overall coculture metabolic activity and showing higher detected rat β-NGF levels at early time points. All experimental groups were maintained under identical culture conditions; therefore, in the unlikely event that absolute NGF concentrations were influenced by serum components and culture supplements, such an influence should have been homogeneous across experimental groups. Thus, the observed differences between groups must be attributed to the scaffold effect, suggesting that anisotropic topographic cues enhance neurotrophic activity without being compromised by click-mediated adhesion. Taken together, these findings support click chemistry as an enabling strategy for the robust assembly of organized biohybrid scaffolds rather than as the sole determinant of the regenerative response observed *in vitro*.

Some limitations for preclinical and clinical translation should be considered. We acknowledge that the present study does not conclusively demonstrate that the improved neural response is directly caused by click chemistry itself. Because scaffold topology, SCs’ presence, and covalent cell-scaffold attachment may all contribute to the observed effects, the specific weight of each factor cannot be fully resolved in the current experimental design. Rather than establishing a direct causal role of click chemistry in neurite outgrowth, this work was designed as a proof of concept to validate that covalent adhesion *via* click chemistry can be used to assemble a stable and biologically functional biohybrid scaffold without impairing its metabolic activity, organization, or capacity to support directed neurite outgrowth *in vitro*. Previous studies in the field of nerve regeneration^44–46^ have demonstrated that one to 2 weeks of *in vitro* culture are sufficient to evaluate key parameters such as neurite outgrowth, cellular alignment, and neurotrophic factor production. Our results confirm that the functionalized scaffolds maintained their ability to support these early regenerative events *in vitro*. However, longer evaluation periods will be required to fully assess the regenerative potential of this strategy *in vivo*, particularly in peripheral nerve injuries involving large gaps, where axonal regeneration typically proceeds at a rate of approximately 1 mm/day. Other important questions, such as the duration and long-term stability of the covalent attachment, also remain to be addressed. Additionally, the functionalization protocol may need to be adapted depending on the specific tissue-engineered construct, as metabolic profiles differ among cell types. Regarding its clinical translation, the first Phase 1/2a clinical trial (NCT04106492) utilizing click chemistry as a cancer therapeutic was completed in 2025, demonstrating its safety in humans.^47^ However, more studies are required to further assess its clinical efficacy and long-term effects. Future directions will focus on expanding the application of click chemistry to additional cell types, enhancing its versatility across diverse target tissues, and assessing the safety and efficacy of the click-linked biohybrids *in vivo*.

## 5 Conclusion

This study shows that biorthogonal click chemistry, specifically SPAAC ligation, enables stable covalent adhesion between SCs and SF microfibers for the development of an artificial BoB *in vitro*, allowing stable cellularization while preserving biological functionality. These results support click chemistry as a useful strategy for improving the robustness and architectural complexity of cell-laden scaffolds in tissue-engineering applications.

## Data Availability

The research data supporting the conclusions of this article will be made available on request.
